# Increase in Net Activity of Serine Proteinases but Not Gelatinases after Local Endotoxin Exposure in the Peripheral Airways of Healthy Subjects

**DOI:** 10.1371/journal.pone.0075032

**Published:** 2013-09-23

**Authors:** Margaretha E. Smith, Steven Bozinovski, Carina Malmhäll, Margareta Sjöstrand, Pernilla Glader, Per Venge, Pieter S. Hiemstra, Gary P. Anderson, Anders Lindén, Ingemar Qvarfordt

**Affiliations:** 1 Lung Immunology Group, Institute of Medicine, Sahlgrenska Academy at the University of Gothenburg, Gothenburg, Sweden; 2 Lung Disease Research Group, Departments of Medicine and Pharmacology, the University of Melbourne, Parkville, Australia; 3 Department of Medical Sciences, University of Uppsala, Uppsala, Sweden; 4 Department of Pulmonology, Leiden University Medical Center, Leiden, the Netherlands; 5 Unit for Lung & Airway Research, Institute of Environmental Medicine, Karolinska Institutet and Lung Allergy Clinic, Karolinska University Hospital, Stockholm, Sweden; University of Bern, Switzerland

## Abstract

We tested the hypothesis that activation of the innate immune response induces an imbalance in the proteolytic homeostasis in the peripheral airways of healthy subjects, towards excess serine or gelatinase proteinase activity. During bronchoscopy, 18 healthy human subjects underwent intra-bronchial exposure to endotoxin and contra-lateral exposure to vehicle. Bronchoalveolar lavage (BAL) samples were harvested 24 or 48 hours (h) later. We quantified archetype proteinases, anti-proteinases, inflammatory BAL cells, and, importantly, total plus net proteinase activities using functional substrate assays. As expected, endotoxin exposure increased the concentrations of polymorphonuclear leukocytes (PMN's) and macrophages, of proteinases and the anti-proteinases tissue inhibitor of metalloproteinase-1, α-1-antitrypsin and, to a lesser extent, secretory leukoproteinase inhibitor, at both time points. Notably, at these time points, endotoxin exposure substantially increased the quantitative NE/SLPI ratio and the net serine proteinase activity corresponding to neutrophil elastase (NE). Endotoxin exposure also increased the total gelatinase activity corresponding to matrix metalloproteinase (MMP)-9; an activity dominating over that of MMP-2. However, endotoxin exposure had no impact on net gelatinolytic activity at 24 or 48 h after exposure. Thus, local activation of the innate immune response induces an imbalance towards increased net serine proteinase activity in the proteolytic homeostasis of the peripheral airways in healthy subjects. Hypothetically, this serine proteinase activity can contribute to tissue remodelling and hypersecretion via NE from PMN's, if it is triggered repeatedly, as might be the case in chronic inflammatory airway disorders.

## Introduction

A well-maintained balance between the local serine proteinases, gelatinases and their inhibitors is believed to be critical for preserving tissue structure and function in the airways, as well as for coordinating the collective immune responses from structural and inflammatory cells to maintain host defence [1], [2], [3], [4], [5], [6], [7]. Thus, the maintenance of the proteolytic homeostasis is important for controlling the release of the archetype chemokine interleukin-8 and the release of even more proteinases [4], [8], [9], [10], [11], [12]. An improved understanding of how the proteolytic homeostasis is maintained in different types of inflammatory settings is also important for the development of pharmacotherapy in acute and chronic airway disorders.

It is known that both polymorphonuclear leukocytes (PMNs) and macrophages are important producers of proteinases and matching anti-proteinases [2], [3], [13], [14] and that there is excess mobilization of PMNs and macrophages in chronic airway disorders displaying detrimental alterations in the proteolytic homeostasis, including chronic obstructive pulmonary disease (COPD) [15], [16] and cystic fibrosis (CF) [17]. Interestingly, there is also evidence of local impairment of anti-proteolytic capacity in community-acquired pneumonia [18].

Even though there is accumulating evidence that the maintenance of the proteolytic homeostasis is involved and altered in several chronic airway disorders, surprisingly little is known about more holistic and functional aspects of the proteolytic homeostasis in the airways of healthy human subjects [1], [2], [3], [19]. One reason for this may be that previous investigators have tended to focus on isolated molecular aspects of the proteolytic homeostasis, without assessing other principal components or total and net proteinase activities in the airways [1], [2], [3], [19], [20]. For example, two very recent studies addressed certain aspects of the serine proteinase neutrophil elastase but there was no corresponding investigation into gelatinases such as matrix metalloproteinase (MMP)-2 and -9 [19], [20]. By limiting their study to samples of induced sputum instead of bronchoalveolar lavage (BAL), the authors of these studies may have missed events unique for the peripheral airways.

In the current study, we tested the hypothesis that activation of the innate immune response induces an imbalance in the proteolytic homeostasis in the peripheral airways, towards excess serine proteinase or gelatinase activity. To test this hypothesis, we conducted a functional plus a quantitative characterization of the proteolytic homeostasis with and without local exposure to the toll-like receptor (TLR)-4 agonist endotoxin. Thus, endotoxin was instilled in a segmental bronchus and its vehicle solution in a contra-lateral one, within each healthy volunteer examined [1]. This intra-bronchial exposure was followed by bronchoalveolar lavage (BAL) 24 or 48 hours (h) later [1]. The proteolytic homeostasis within the peripheral airways was subsequently assessed in a multitude of ways, including the quantification of inflammatory cells, archetype proteinases, anti-proteinases and, importantly, the total and the net proteinase activities. In doing so, we obtained evidence that activation of the innate immune response induces an imbalance in the proteolytic homeostasis of the peripheral airways in healthy human subjects, towards increased net serine proteinase activity.

## Materials and Methods

### Design

All participating subjects gave oral and written informed consent, in accordance with the Helsinki Declaration. The appropriate ethical permission (No. 618-02; T065-04 and T683-07) was obtained from the Ethical Review Board for studies on humans in Gothenburg.

Our study was performed at the Clinical Section of Respiratory Medicine at Sahlgrenska University Hospital in Gothenburg, Sweden. Healthy human volunteers were recruited through local advertising. The clinical part of the study took place from Nov 11, 2003 to Jan 14, 2005. All samples were processed in the involved laboratories during 2006 to 2008.

During the first clinical visit, the medical history was recorded and physical examination, spirometry and electrocardiogram were performed. Current or past smoking, a history of allergy or atopy and any regular medication constituted exclusion criteria, with the exception of oral contraceptives.

All included subjects had a normal ventilatory lung capacity defined as forced expiratory volume during one second (FEV_1_) >80% of predicted value, as well as normal findings in their electrocardiogram and physical status. Bronchoscopies were performed during the second and third visit, separated by an interval of either 24 or 48 h. The allocation of subjects to either time point was performed on a practical basis, set by the complex hospital logistics. Endotoxin exposure took place at visit two (bronchoscopy I, B.I) and BAL was conducted at visit three (bronchoscopy II, B.II).

### Blood samples

Immediately before B.I and B.II, venous blood was drawn and sent to the laboratory of Sahlgrenska University Hospital (accredited by the Swedish Board of Accreditation and Conformity Assessment, SWEDAC) for analysis of haemoglobin, total blood leucocytes (LPC) and differential leukocyte counts as well as C-reactive protein (CRP). Plasma and serum from additional blood samples was separated, frozen and stored at −70°C pending further analyses.

### Bronchoscopies, endotoxin exposure and bronchoalveolar lavage

All bronchoscopies were performed trans-orally by one very experienced bronchoscopist who is a board-certified Clinical Specialist in Respiratory Medicine (Smith, M.E.) with the subject in supine position. We utilized a slightly modified version of a previously published endotoxin exposure protocol [1], [21]. Briefly, ketobemidonhydroklorid (2.5 to 7.5 mg depending on clinical conditions) was given as pre-medication, followed by a nebulized local anaesthesia sprayed into the oropharynx (xylocaine 10 mg/dose, 3×2 doses). Additional local anaesthesia was given as needed through the bronchoscope (xylocaine 20 mg/mL, up to 14 mL). Endobronchial photographs were taken bilaterally during B.I and B.II to ensure that the BAL sampling was performed in the same bronchial segments that had been exposed to endotoxin or vehicle.

The following procedures were applied for B.I: A balloon-tipped catheter was inserted through the bronchoscope, placed in a bronchial segment (either right middle lobe or lingula) and inflated with air to seal off the chosen segment proximally before challenge. The instillation of vehicle (10 mL of 0.9% phosphate-buffered saline, PBS) and inflation of a small volume of air (10 mL) into the bronchial segment was then conducted. The bronchoscope was subsequently retracted and transferred to the corresponding segment in the contra-lateral lung. The sealing off-procedure was followed by instillation of endotoxin (USP reference standard endotoxin from *Eschericia coli 0113*: H10, from USP, Rockville, MD, USA) and inflation of air (10 mL). Endotoxin was given in a dose proven both efficacious and clinically safe (4 ng/kg diluted with PBS up to 10 mL of fluid) [1]. Finally, the bronchoscope was subsequently retracted and the head end of the operating table was immediately elevated 30° with the subject in place during one hour (h) to minimize spread of instilled fluid from the challenged segments.

For B.II, the following procedures were applied: This bronchoscopy was performed at either 24 or 48 h after B.I. The bronchial segments in each lung, exposed to either endotoxin or vehicle, were re-identified, the bronchoscope was wedged in each of these segments and BAL was performed (3×50 mL of PBS at 37°C), starting with the vehicle-exposed segment. After the subsequent aspiration of each 50 mL aliquot, the entire harvested yield was pooled in a siliconized glass container. This BAL sample was immediately transported on ice to our research laboratory. The recovered volume was then measured and the fluid filtered for retention of mucus and cell debris. After this, the filtered BAL sample was centrifuged (300×G, 10 min at 4°C). The cell-free BAL fluid (i.e. the centrifugation supernatant) was then separated and frozen at –80°C until further analysis.

The cell pellet from the centrifugation was re-suspended in PBS and cell viability was determined using trypan blue exclusion as measure. The total cell count was determined utilizing a haemocytometer (Bürker chamber). The differential counts for BAL cells were performed utlizing stained cytospin preparations after counting of 2×300 cells using a conventional light microscope (Axioplan® 2, Carl Zeiss™, Jena GmbH, Eching, Germany).

### Symptom assessment

Clinical symptoms were recorded immediately prior to B.I and B.II, and at three different time points after each bronchoscopy, using a questionnaire (supporting information).

### Immunocytochemistry

To prepare for immunocytochemistry (ICC), the BAL cells were fixed (2% formaldehyde, 30 min) and washed twice in buffer prior to making the cytospin preparations. Air dried samples were stored in –83°C until further use. To avoid unspecific binding, samples were treated with 10% donkey serum (Jackson™ ImmunoResearch laboratories Inc, West Grove, PA, USA). Endogenous biotin was blocked using Biotin Blocking System (DAKO™ Corporation, Glostrup, Denmark). The ICC slides were further incubated with a polyclonal goat anti-human MMP-9 antibody (R&D Systems™ Europe, Abingdon, Oxfordshire, UK) during 1 h. As secondary antibody, a biotinylated F(ab‚)_2_ fragment donkey anti-goat IgG (Jackson ImmunoResearch™ laboratories Inc) was used, followed by alkaline phosphatase-conjugated streptavidin (DAKO). Bound antibodies were visualised with Vector Red Alkaline Substrate Kit (Vector™ Laboratories, Inc. Burlingame, CA, USA). Mayer's Hematoxylin (Sigma™, St Louis, MO, USA) was used for counter-staining. The stained ICC samples were assessed in a blinded fashion under a light microscope (Axioplan® 2) at a magnification of ×400.

### Quantification of soluble proteinases and anti-proteinases in BAL fluid

Examining cell-free BAL fluid, we quantified the archetype gelatinases matrix metallo-proteinase (MMP)-2 and MMP-9 [22], [23], respectively, using both zymography [14] and enzyme-linked immunosorbent assay (ELISA, Quantikine®, R&D Systems™ Europe). We detected MMP-9 in all ELISA samples but MMP-2 was not detectable in some samples. These particular samples were assigned a value of 0.39 ng/mL, corresponding to half that of the lower detection limit (0.78 ng/mL) of the utilized assay. Examining cell-free BAL-fluid, we also quantified human neutrophil lipocalin (HNL), that forms an extracellular complex with MMP-9 [24], [25], utilising a solid-phase, double-antibody radioimmunoassay as previously described [26]. Finally, when examining cell-free BAL fluid, we also quantified serine proteinase neutrophil elastase (NE) [4], a potent secretagogue, using a solid-phase ELISA developed at the Dept. of Pulmonology, Leiden University Medical Center (LUMC), the Netherlands [27]. Notably, this assay allows measurement of both free NE and NE bound to its inhibitors.

When analysing cell-free BAL fluid, we also quantified the concentrations of endogenous anti-proteinases including tissue inhibitor of metalloproteinase (TIMP-1), that binds and inactivates MMP-9 [28], [29], and secretory leukoproteinase inhibitor (SLPI), that counteracts NE [28], [29] (Quantikine®, R&D Systems™ Europe). In a corresponding manner, we also quantified the concentrations of α-1-antitrypsin (AAT), that inhibits NE [30] using ELISA (ImmuChrom™, Heppenheim, Germany).

### Determination of net gelatinase and serine proteinase activity

These measurements were based upon functional substrate assays. The net gelatinase activity in BAL fluid (BALF) was measured as previously described [14] to functionally assess excess proteolytic activity generated by gelatinases. Briefly, the specific fluorescence conjugated gelatine substrate (Molecular Probes™, Quantum Scientific, Murarrie, Queensland, Australia) was incubated at room temperature with cell-free BAL fluid during 16 h. The fluorescence intensity of the digested substrate was measured in a microplate reader (Victor II, Wallac, Melbourne, Australia) to detect quantitative differences in activity and the result was expressed in arbitrary units.

The net serine proteinase activity was measured as previously described [31], [32] to functionally assess excess proteolytic activity generated by serine proteinases. Briefly, a specific and well-documented commercially available substrate (N-methoxysuccinyl-Ala-Ala-Pro-Val p-Nitroanalide # 4765, supplied by Sigma™) was diluted in a reaction buffer and incubated (16 h, at room temperature) with cell-free BAL fluid. Degraded peptide was detected by measuring the relative absorbance intensity at 405 nm, using a microplate reader (Victor II), and the result was expressed in arbitrary units.

### Statistical considerations

The SPSS 14.0 (SPSS Inc™, Chicago, Ill, USA) software package was used for the statistical analyses. Since normal distribution of results could not be proven, due to the limited sample sizes, data are presented in a non-parametric manner, as median and individual values with range. For comparisons between groups, paired (Wilcoxon Signed rank test) or unpaired (Mann Whitney U-test) non-parametric tests were used. As appropriate, paired comparisons were used for matched samples obtained from the same subjects (samples from endotoxin- versus vehicle-exposed bronchial segments). Unpaired comparisons of the effect of endotoxin exposure over time (samples from different subjects) were made by calculating the difference between results from the vehicle- and endotoxin-exposed segment within each subject, at each time point, followed by a comparison of these differences between 24 and 48 h. For correlations between individual data in different groups Spearmańs two-tail rank correlation test was applied. *p*-values <0.05 were accepted as statistically significant. Repeated comparisons were not compensated for, to avoid an overly conservative outcome of the analysis.

## Results

### Study performance and clinical symptoms

Nineteen healthy human volunteers (21–30 years of age, 10 male and 9 female) completed the study protocol. One female subject in the 48 h group experienced transient nausea and vomited immediately after B.I. Due to the risk of micro-aspiration influencing the BAL yield, this subject was excluded. Consequently, we finally included 11 subjects at 24 h and 7 subjects at 48 h. Symptoms of side effects associated with B.I, including the endotoxin exposure, were mild and transient (Table S1 in File S1).

### Blood samples

In two subjects, data could not be retrieved for technical reasons. For all other subjects, the results at baseline prior to B.I were normal (Table S2 in File S1). In association with B.II, at 24 h but not at 48 h after endotoxin exposure, CRP levels, LPC and PMN counts were slightly and consistently increased, compared with B.I.

### Recovery of BAL, viability and concentrations of inflammatory cells

While the recovery of BAL volume from the endotoxin-exposed bronchial segments was comparable with that of the vehicle-exposed segments, the cell viability was significantly higher in BAL samples from the endotoxin-exposed segments at both time points (Table S3 in File S1). There were no apparent gender differences in either recovery or cell viability (data not shown).

**Figure 1:**
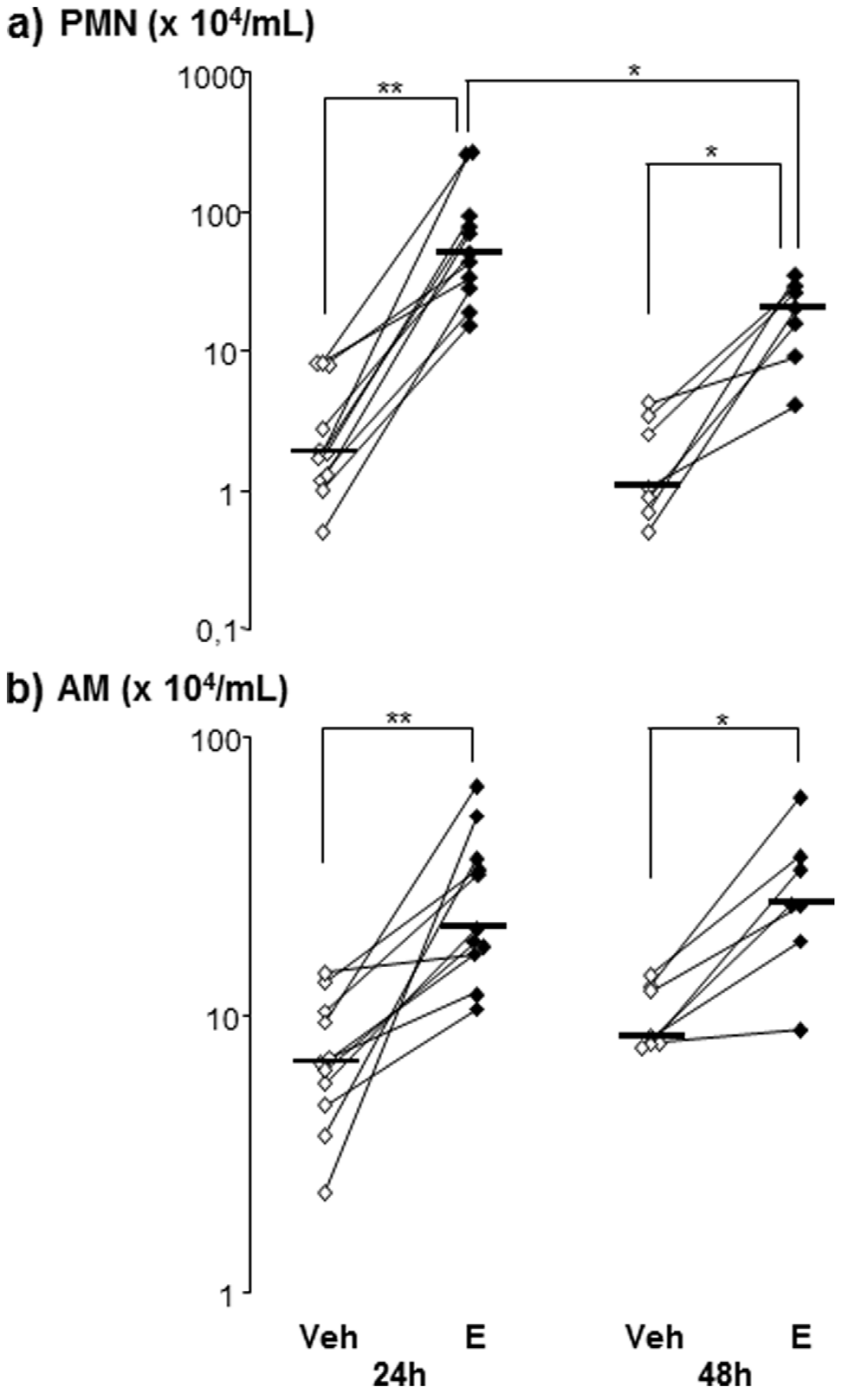
Inflammatory cells in bronchoalveolar lavage (BAL) samples harvested from vehicle (Veh)- and endotoxin (E)-exposed bronchial segments in healthy subjects. Panels show concentrations of a) polymorphonuclear leucocytes (PMN's) and b) alveolar macrophages (AM) in BAL samples harvested at 24 (n = 11) and 48 (n = 7) hours (h) after exposure. Median and individual values are shown. Asterisks indicate statistically significant differences between BAL samples from endotoxin- and vehicle-exposed segments (paired test), as well as statistically significant differences between 24 and 48 h in endotoxin-exposed segments (un-paired test) (* = p<0.05, ** =  p<0.01).

The total concentration of all inflammatory cells was considerably higher in BAL samples from endotoxin-exposed segments than in those from the vehicle-exposed segments at 24 h [80 (47–353) *versus* 13 (6–26)×10^4^ cells/mL, p<0.05] and 48 h [66 (20–127) *versus* 15 (11–23)×10^4^cells/mL, p<0.05]. This was also true for the concentrations of PMNs in BAL samples (Figure 1), which were on average a 32-fold (4–143) higher in the endotoxin-exposed segments at 24 h. The BAL macrophage concentrations were also substantially increased (Figure 1) and were on average three-fold (1.1–23) higher in the endotoxin-exposed segments at both time points. The endotoxin-induced changes in the distribution of PMNs and macrophages in BAL samples are presented separately (Table 1).

**Table 1:** Differential count for polymorphonuclear leukocytes (PMN) and alveolar macrophages (AM) in bronchoalveolar lavage (BAL) samples harvested from vehicle (Veh)- and endotoxin (E) -exposed bronchial segments in healthy subjects.

Cell (%)	24h Veh	24h E	48h Veh	48h E	
PMN	24 (4–39)	61 (40–76)^a^	8 (4–28)	27 (20–40)^b,c^	
AM	52 (37–70)	28 (15–29)^a^	60 (55–75)^d^	44 (37–49)^b,c^	

*Footnote.* The BAL samples were harvested 24 (n = 11) and 48 (n = 7) hours (h) after exposure. Median values (range) are shown. a) E versus (*vs).* Veh at 24 h; p<0.01 (paired test), b) E *vs.* Veh at 48 h; p<0.05 (paired test), c) E 24h *vs.* 48 h; p<0.01 (un-paired test), d) Veh 24 h *vs.* 48 h; p<0.05 (un-paired test).

### Zymography of cell-free BAL fluid

The zymography analysis identified clearly detectable “bands” of gelatinolytic activity in BAL fluid, regardless of endotoxin exposure, mainly at 72 kD and 92 kD, corresponding to MMP-2 and MMP-9, respectively (Figure S1 in supporting information). There was also a third band at 110-130 kD, corresponding to the size of the complex of MMP-9 and HNL [24], [33]. Moreover, densitometry revealed much higher intensity corresponding to the MMP-9 bands in comparison with the MMP-2 bands, irrespectively of endotoxin exposure (Table 2). To address the relative contribution to the total gelatinolytic activity by the two MMPs respectively, we calculated a ratio between the band intensities corresponding to MMP-9 and MMP-2 in each individual subject. Samples with no detectable activity (n = 5, all from vehicle-exposed segments) were assigned the value 1, to enable the calculation of ratios. The median intensity of the MMP-9 bands was much (8-54 fold) higher than that of the MMP-2 bands from the different compartments at both time points (Table 2).

**Table 2:** Total gelatinase activity for matrix metalloproteinase (MMP)-9 and MMP-2 in cell-free bronchoalveolar lavage (BAL) fluid samples harvested from vehicle(Veh)- and endotoxin(E)-exposed bronchial segments in healthy subjects.

	24h Veh	24h E	48h Veh	48h E	
**MMP-9** (Units)	69,773* (13,852–159,397)	135,976** (39,650–216,747)	21,483* (4,140–70,548)	114,835* (20,858–316,992)	
**MMP-2** (Units)	772 (0–2,403)	8,562 (1465–66,211)	945 (0–2,473)	7,644 (760–52,361)	
**Ratio MMP-9/MMP-2**	54 (7–159,397)	12 (2–55)	34 (9–37,719)	8 (4–104)	

*Footnote.* Zymographic activity of MMP-9 and MMP-2 measured using densitometry and expressed in arbitrary units. The BAL fluid samples were harvested 24 (n = 11) or 48 (n = 7) hours (h) after exposure to vehicle (Veh) or endotoxin (E). Individual ratios for MMP-9 and MMP-2 activity for each segment were calculated and are shown in the bottom row. All values are shown as medians with range. Asterisks indicate statistically significant differences between the total activities of MMP-9 and MMP-2, for endotoxin- and vehicle-exposed segments, respectively (* = p<0.05; ** = p<0.01, paired test).

### Soluble proteinases and anti-proteinases in cell-free BAL fluid

**Figure 2:**
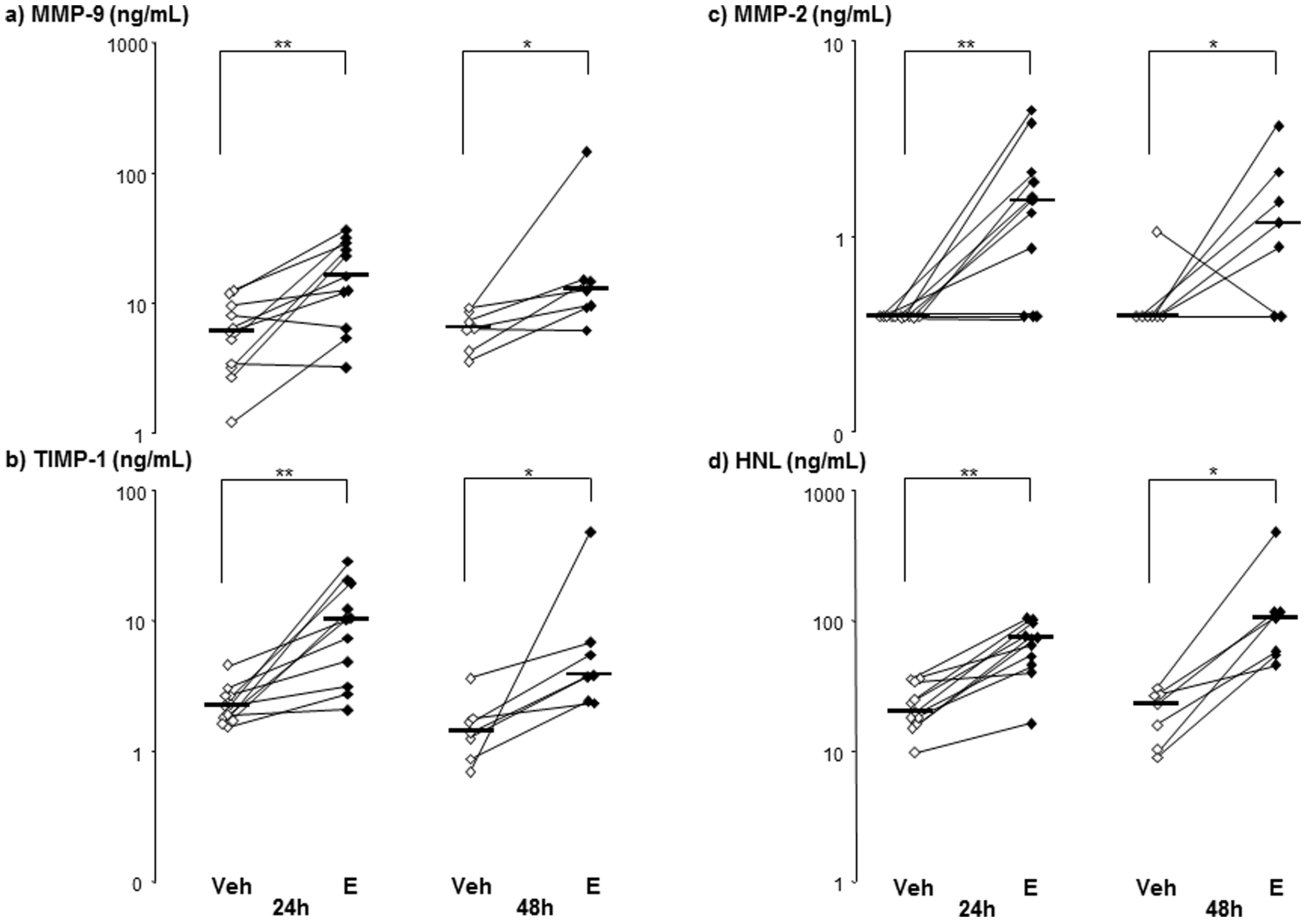
Gelatinases and their inhibitors in bronchoalveolar lavage (BAL) samples harvested from vehicle (Veh)- and endotoxin (E)-exposed bronchial segments in healthy subjects. Panels show protein concentrations of a) matrix metalloproteinase (MMP)-9, b) tissue inhibitor of metalloproteinase (TIMP)-1, c) MMP-2 and d) human neutrophil lipocalin (HNL) in cell-free BAL fluid samples harvested at 24 (n = 11) and 48 (n = 7) hours (h) after exposure. Median and individual values are shown. Asterisks indicate statistically significant differences between BAL fluid samples from endotoxin- and vehicle-exposed segments (* = p<0.05, ** =  p<0.01, paired test).

**Figure 3:**
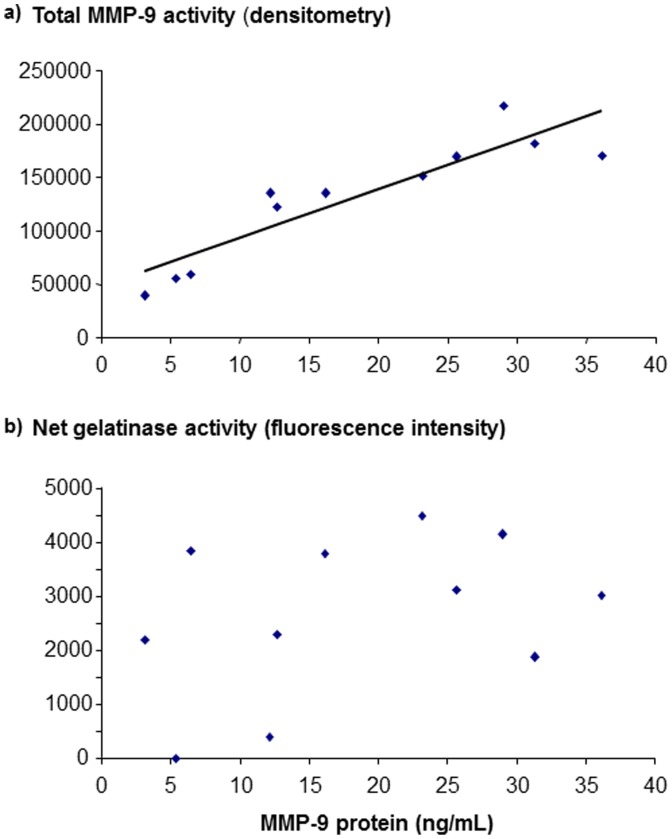
The correlations between the concentrations of free soluble matrix metalloproteinase (MMP)-9 protein and total and net gelatinase activity, respectively, in cell-free bronchoalveolar lavage (BAL) fluid samples harvested 24 hours after intra-bronchial endotoxin-exposure in healthy subjects (n = 11). Panels show a) the correlation (rho = 0.94, p<0.01) for the concentration of free soluble MMP-9 protein and total MMP-9 activity and b) the correlation (rho = 0.34; p = 0.31) for the concentration of free soluble MMP-9 protein and net gelatinase activity. Individual values are shown. a.u. =  arbitrary units.

The concentrations of MMP-9, its inhibitor TIMP-1, MMP-2 and HNL are shown in Figure 2. The median concentrations of the two metalloproteinases (MMP-9, MMP-2), TIMP-1 and HNL were higher in cell-free BAL fluid from the endotoxin-exposed bronchial segments, compared with the vehicle-exposed segments at both time points. Moreover, in the samples from the endotoxin-exposed segments, the concentrations of MMP-9, MMP-2 and TIMP-1 were higher at 24 than at 48 h but this difference was not statistically significant. The total gelatinase activity (densitometry) and the concentration of soluble MMP-9 protein (ELISA) displayed a strong correlation, irrespective of time point and compartment (Figure 3a). In contrast, there was no corresponding correlation for the net gelatinase activity and the concentration of MMP-9 (Figure 3b).

**Figure 4:**
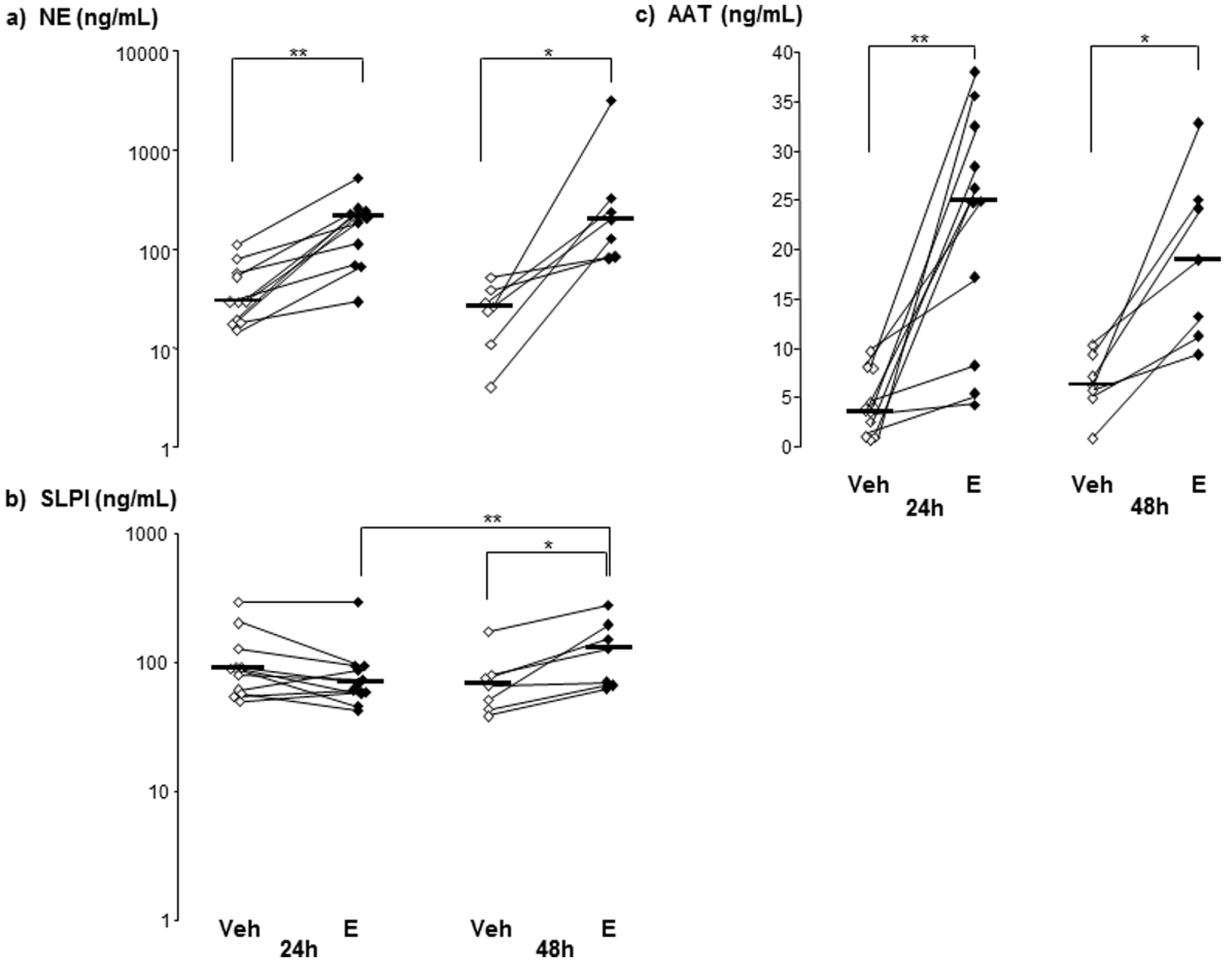
Neutrophil elastase (NE) and its inhibitors in bronchoalveolar lavage (BAL) samples harvested from vehicle (Veh)- and endotoxin (E)-exposed bronchial segments in healthy subjects. Panels show protein concentrations of a) NE, b) secretory leukoproteinase inhibitor (SLPI) and c) α-1-antitrypsin (AAT) in cell-free samples harvested at 24 (n = 11) and 48 (n = 7) hours (h) after exposure. Median and individual values are shown. Asterisks indicate statistically significant differences for samples from endotoxin- and vehicle-exposed segments (paired test), as well as statistically significant differences between 24 and 48 h (un-paired test) samples from endotoxin-exposed segments (* = p<0.05, ** =  p<0.01).

**Figure 5:**
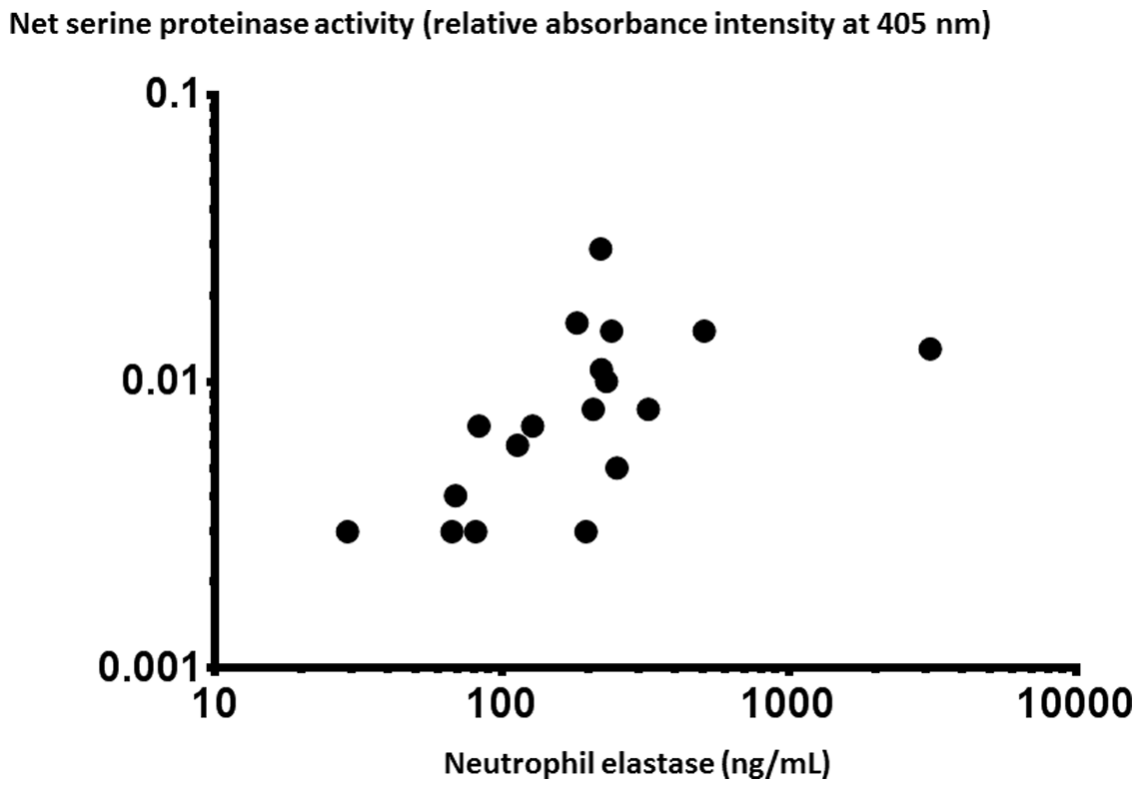
The correlation (rho = 0.66; p = 0.003) between the concentration of free soluble neutrophil elastase protein (NE) and net serine proteinase activity in cell-free bronchoalveolar lavage (BAL) fluid harvested after intra-bronchial instillation of endotoxin in healthy subjects. Samples were harvested 24 (n = 11) and 48 (n = 7) hours (h) after the instillation.

The concentrations of NE, SLPI and AAT are shown in Figure 4. The concentration of NE followed the patterns of the two gelatinases (MMP-9 and MMP-2); in this case with a (2- to 8-fold) higher median concentration in cell-free BAL fluid samples from the endotoxin-exposed bronchial segments compared with the vehicle-exposed ones and this finding was present both at 24 and 48 h after exposure. The same pattern was observed for AAT, one of the two serine proteinase inhibitors investigated. In contrast, the concentrations of SLPI, the other of the two serine proteinase inhibitors investigated, did not markedly differ in BAL fluid samples from endotoxin- and vehicle-exposed segments at 24 h, whereas SLPI concentrations were significantly higher in samples from the endotoxin-exposed segments at 48 h. The median SLPI concentration in BAL fluid samples from the endotoxin-exposed segments was higher at 48 than at 24 h. Furthermore, the net serine protease activity (relative absorbance intensity) and the concentration of soluble NE (ELISA) displayed a relatively strong correlation in BAL fluid from endotoxin-exposed segments (Figure 5). In addition, the median (range) individual NE/SLPI ratio was clearly and consistently higher in endotoxin-exposed than in vehicle-exposed segments at 24 h [3.53 (0.23–8.83) *versus* 0.36 (0.05–1.26), p = 0.001, n = 11] and at 48 h [1.14 (0.42–24.95) *versus* 0.32 (0.14–0.76), p = 0.03, n = 7] after endotoxin exposure.

### Immunocytochemistry of BAL cells

The ICC staining for MMP-9 indicated intracellular localization of this gelatinase in PMNs mainly, at both time points (Table 3). The proportion of PMNs that stained positively for MMP-9, as well as the staining intensity, was higher in BAL cells from the endotoxin-exposed segments compared with the vehicle-exposed ones, at both 24 and 48 h (Table 3; Figure S2 in File S1). In the endotoxin-exposed segment, we found relatively more BAL macrophages that stained positively for MMP-9, compared with the vehicle-exposed ones, and this pattern appeared more pronounced at 48 compared with 24 h (Table 3). Some of these macrophages contained engulfed PMN's (Figure S2c in File S1). Only a negligible proportion of cells other than macrophages displayed positive ICC staining for MMP-9 (data not shown).

**Table 3:** Immunoreactivity for matrix metalloproteinase (MMP)-9 protein in polymorphonuclear leukocytes (PMN) and in alveolar macrophages (AM), respectively, in bronchoalveolar lavage (BAL) samples harvested from vehicle (Veh) - and endotoxin (E)-exposed bronchial segments in healthy subjects.

	24h	24h	48h	48h
Exposure	Veh	E	Veh	E
All (% positive cells)	6.2 (3–20)	58 (33–69)*	9 (1.5–15)	26 (12–39)*
PMN (% positive cells)	6 (3–19)	56 (29–68)*	6 (1.2–13)	19 (8–33)*
AM (% positive cells)	0 (0.2–0.5)	0.4 (0–0.8)	0.2 (0–1.2)	1.5 (0.5–2.2)*

*Footnote.* Immunoreactivity assessed using immunocytochemistry of BAL cells on cytospin slides. Samples were harvested 24 (n = 6) and 48 (n = 7) hours (h) after exposure. Data presented as median values (range), in percent of respective cell type. Asterisks indicate statistically significant differences between samples from endotoxin- and vehicle-exposed segments (* = p<0.05, paired test).

### Net serine and gelatinase proteinase activity in BAL fluid

**Figure 6:**
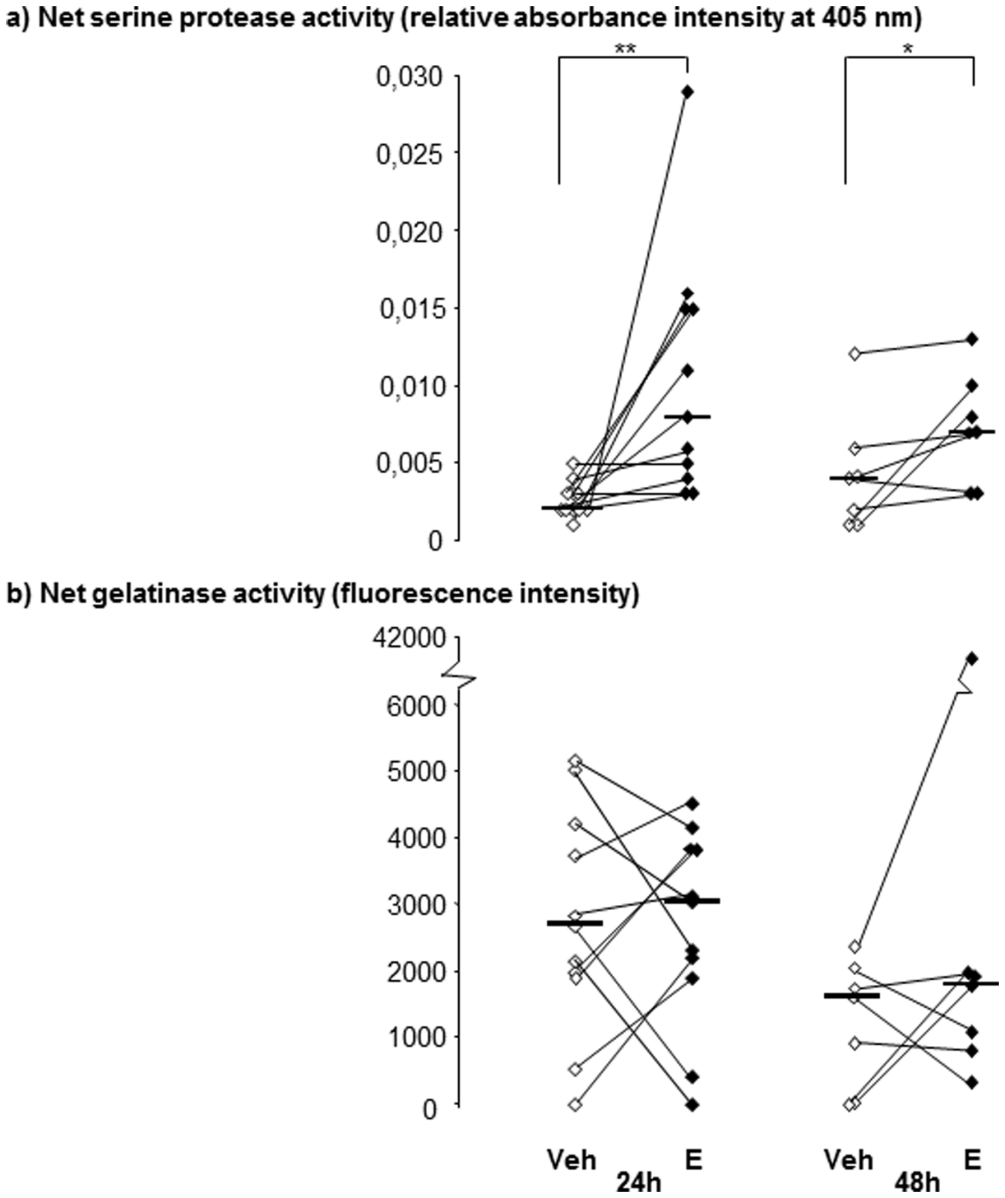
The net proteolytic activity in bronchoalveolar lavage (BAL) samples from healthy subjects. The net proteolytic activity was assessed in cell-free samples for a) serine proteinases and b) gelatinases. Samples were harvested at 24 (n = 11) and 48 (n = 7) hours (h) after local instillation of vehicle (Veh) and endotoxin (E) in contra-lateral bronchial segments within each subject. Median and individual values are shown. Asterisks indicate statistically significant differences between samples from endotoxin- and vehicle-exposed bronchial segments (* = p<0.05, ** =  p<0.01, paired test).

There was a higher net serine proteinase activity in the BAL fluid from endotoxin- than in the vehicle-exposed segments at 24 and 48 h after exposure (Figure 6a). There was also a tendency towards the endotoxin-induced increase in net serine proteinase activity being greater at 24 h than at 48 h (Figure 6a). The median net gelatinolytic activity, on the other hand, did not differ significantly with reference to exposure or time point (Figure 6b).

## Discussion

With this study, we tested the hypothesis that activation of the innate immune response induces an imbalance in the proteolytic homeostasis in the peripheral airways. We found that after activation of the innate immune response with the TLR-4 agonist endotoxin, healthy human subjects displays an imbalance in the proteolytic homeostasis in the peripheral airways, towards an increase in the *net* activity for serine proteinases but not gelatinases.

We think that the identification of net serine proteinase activity as a discriminating factor in the peripheral airways was facilitated by the design of our study, including non-smokers only and a protocol with limited and well-defined exposure to endotoxin. The fact that our protocol controlled for vehicle, bronchoscopy and time within each subject is probably important as well.

The serine proteinase substrate N-methoxysuccinyl-ala-ala-pro-val-p-nitroanilide is well-documented in the literature [18], [20], [31], [34]. Using this substrate, we found a clear and statistically significant correlation between net serine proteinase activity and NE concentration in cell-free BAL fluid. This supports the idea that the imbalance in proteolytic homeostasis towards a sustained net serine proteinase activity corresponds at least partially to the archetype serine proteinase NE. In further support of this idea, we found a matching increase in the ratio for NE and its archetype anti-proteinase SLPI at both 24 and 48 h after exposure. In spite of these findings, however, we cannot rule out that additional serine proteinases are involved in the peripheral airways of healthy subjects. Moreover, there was no increase in net gelatinase activity after endotoxin exposure in our study, neither at 24 or 48 h after exposure. Thus, in healthy subjects, activation of the innate immune response does not induce an imbalance towards excess gelatinase activity in the proteolytic homeostasis in the peripheral airways.

On a hypothetical basis, it could be argued that the induced and sustained increase in serine proteinase activity that we demonstrate relates to the dose and route of administration of endotoxin. However, there is published evidence supporting an increase in serine proteinases caused by bacterial stimuli, obtained in studies with different doses and routes of administration. These studies include the utilization of inhaled as well as intra-bronchial administration of endotoxin; the latter as in our current study [3], [19], [20].

Notably, our current demonstration of an endotoxin-induced imbalance towards increased net serine proteinase activity may have bearing not only for gram-negative but also for gram-positive infections. This is because local impairment of “anti-neutrophil elastase capacity” has been demonstrated in patients with community-acquired pneumonia, predominantly caused by Streptococcus pneumonie [18].

Indeed, the serine proteinase inhibitor AAT was increased within 24 h after endotoxin exposure in our study; the time point associated with the highest net serine proteinase activity. Since BAL samples primarily reflect conditions in the peripheral airways, and presumably the alveoli as well, this observation argues against AAT being sufficient for protecting against excess NE activity at this level [4], [28]. This is because the increase in AAT at 24 h after endotoxin exposure lacked association with an unaltered or reduced net serine proteinase activity. However, the increase in AAT did follow the increase in PMN concentrations, which is compatible with PMNs constituting important sources of AAT [35], [36].

As expected, our results confirm that the immune response to endotoxin in the peripheral airways of healthy subjects include a very pronounced accumulation of PMNs and a less pronounced accumulation of macrophages. Whereas the PMN accumulation tended to fade the macrophages peaked at 48 h after exposure. Interestingly, we noted engulfment of seemingly apoptotic PMNs in macrophages at 48h as well, suggesting an effective resolution of the innate immune response, in line with previous studies on induced sputum after exposure to inhaled endotoxin in healthy airways [19].

We think that the fact that there was a decline in BAL PMN concentrations from 24 to 48 h in vehicle-exposed segments, mirroring the endotoxin-exposed segments, can be attributed to our exposure model. In this model, BAL samples from vehicle-exposed bronchial segments reflect the sum of “inherent conditions” plus the “un-specific response” to vehicle *per se*, to bronchoscopy (B.I), as well as the response to some “spill-over” of endotoxin from the contra-lateral bronchial segment at each time point. In our study, we thus controlled for vehicle, bronchoscopy and timing within each subject.

The increase in absolute MMP-9 concentrations after endotoxin exposure followed the corresponding increase in PMN and NE concentrations at 24 h after exposure in our study. The same pattern was evident for the ICC signal of MMP-9; a signal predominantly observed in PMNs. However, as opposed to the endotoxin-induced increase in MMP-9 concentrations, the corresponding increase in NE concentrations remained virtually unaltered at 48h. This also indicates a more sustained innate immune response for healthy, non-smoking subjects in terms of NE compared with MMP-9.

The concentrations of HNL tended to be higher at 48 h in BAL samples from endotoxin- than from vehicle-exposed segments, a pattern that is clearly different from what we observed for PMNs, MMP-9 and NE. The mechanistic rationale for this observation remains uncertain but may relate to the fact that HNL, MMP-9 and NE all originate from different types of intracellular granulae [22], [24], [37], [38], [39].

We found that the time course of alterations in TIMP-1 concentrations relatively well followed that of MMP-9 concentrations, although the concentration of TIMP-1 declined somewhat more than that of MMP-9 at 48 h. This is of course compatible with the proteolytic homeostasis being critical to preserve structure and function of the airways, to avoid the excess proteinase activity present in inflammatory disorders like COPD and CF [15], [16], [17], [22], [29], [30], [38].

Our assessment of total gelatinase activity (using zymography) suggests that MMP-9 is the quantitatively dominant one among archetype gelatinases in the peripheral airways of healthy subjects, with or without activation of the innate immune response. However, our quantification of the absolute MMP-9 concentrations (using ELISA) and the functional assessment of net gelatinase activity (using a functional substrate assay) did not verify a clear association of these two outcomes. Unexpectedly, our quantification of net gelatinase activity showed that this functionally decisive outcome was comparable in BAL samples with or without endotoxin exposure. Thus, our results suggest that the net gelatinase activity is relatively stable over time with or without activation of the innate immune response. At a first glance, this finding may appear contradictory to previous speculations that the excess release of MMP-9 is involved in tissue remodelling in severe asthma and COPD [40], [41], [42]. We think that the key to understand these observations is the fact that a certain increase in gelatinase concentration does not warrant a corresponding increase in *net* gelatinase activity. The *net* gelatinase activity is likely to be the critical functional assessment, reflecting a balance between gelatinases and the matching anti-proteinases. Possibly, the method (ELISA) that we utilized to detect the endotoxin-induced increase in MMP-9 concentrations does not strictly reflect the finally processed MMP-9; the induced release of pro-MMP-9 molecules that are not enzymatically active may also have been detected. It could also be that the lack of an endotoxin-induced increase in net gelatinase activity reflects physiological conditions rather than pathology. To further evaluate these matters, our current study design should be applied in new clinical studies.

In conclusion, the key finding of the current study is that activation if the innate immune response with a TLR-4 agonist induces an imbalance towards increase in net activity of serine proteinases but not gelatinases in the peripheral airways. The utility of this imbalance remains unclear since, hypothetically, it can contribute to pathology in terms of tissue remodelling and destruction, to the stimulation of pro-inflammatory cytokines, other proteinases and gland secretion via NE from PMN's, if it is triggered repeatedly [43]. Clearly, the demonstrated increase in *net* serine proteinase activity may have implications for the development of pharmacotherapy targeting endotoxin-related airway disease. New clinical studies in acute and chronic airway disorders are therefore warranted.

## Supporting Information

File S1 **Table S1.** Symptom assessment after the first bronchoscopy (B.I) in 18 healthy subjects. n =  number of subjects reporting each symptom. **Table S2.** Blood concentrations of C-reactive protein (CRP), total leukocytes (LPC) and polymorphonuclear leukocytes (PMN's) at baseline prior to bronchoscopy I (B.I), and 24 or 48 hours (h) after intra-bronchial endotoxin exposure in healthy subjects. **Table S3**. Recovery of and cell viability in bronchoalveolar lavage (BAL) fluid samples harvested after intra-bronchial exposure to vehicle (Veh) and endotoxin (E), respectively, in 18 healthy subjects. Samples were harvested either 24 (n = 11) or 48 (n = 7) hours after the exposure. **Figure S1.** Photo of representative zymography gel, based upon analysis of cell-free bronchoalveolar lavage (BAL) fluid samples harvested after intra-bronchial exposure to vehicle (no arrow) and endotoxin (arrow), respectively, at 24 hours (h, black arrow) or 48 h (open arrow) in healthy subjects. The samples display three bands representing different molecular weights, corresponding to matrix metalloproteinase (MMP)-2 (72 kD), MMP-9 (92 kD) and a complex bound form of MMP-9 (130 kD). *Two lanes from the same subject. **Figure S2.** Photomicrograph of representative cytospin slides showing bronchoalveolar lavage (BAL) cells containing intracellular matrix metalloproteinase (MMP)-9 harvested after intra-bronchial exposure to endotoxin and vehicle, respectively, in healthy subjects. Immunoreactivity to MMP-9 is red and polymorphonuclear (PMN) cells with positive staining are indicated with arrows. Panels show samples from segments exposed to a) endotoxin and b) vehicle from the same subject at 24 hours (h) and c) endotoxin at 48 h after exposure from another subject. In panel c, the PMN indicated by an arrow is engulphed by a macrophage.
(DOCX)
