# Supplementary material for: Increase in Net Activity of Serine Proteinases but Not Gelatinases after Local Endotoxin Exposure in the Peripheral Airways of Healthy Subjects
Source: PLoS One. 2013 Sep 23;8(9):e75032. doi: 10.1371/journal.pone.0075032 (PMC3781029; doi:10.1371/journal.pone.0075032)

**Supporting information**

**Increase in net activity of serine proteinases but not gelatinases after local endotoxin exposure in the peripheral airways of healthy subjects**

**Margaretha E. Smith^1^; Steven Bozinovski^2^; Carina Malmhäll^1^; Margareta Sjöstrand^1^; Pernilla Glader^1^; Per Venge^3^; Pieter S. Hiemstra^4^; Gary P. Anderson^2^; Anders Lindén^1,5^, Ingemar Qvarfordt^1^.**

1) Lung Immunology Group, Institute of Medicine, Sahlgrenska Academy at the University of Gothenburg, Gothenburg, Sweden.

2) Lung Disease Research Group, Departments of Medicine and Pharmacology, the University of Melbourne, Parkville, Australia

3) Department of Medical Sciences, University of Uppsala, Uppsala, Sweden

4) Department of Pulmonology, Leiden University Medical Center, Leiden, the Netherlands.

5) Unit for Lung & Airway Research, Institute of Environmental Medicine, Karolinska Institutet and Lung Allergy Clinic, Karolinska University Hospital, Stockholm, Sweden

**METHODS**

**Symptom Assessment**

Clinical symptoms were recorded at baseline, prior to B.I and B.II, as well as at 1, 3 and 12 h after each bronchoscopy, using a questionnaire. Both systemic (malaise, headache, fever) and pulmonary (cough, chest pain, mucus) symptoms were assessed and their severity were quantified by each included subject (0=none, 1=mild, 2= moderate, 3=severe).

**RESULTS**

**Table S1.**

| **Symtoms & signs** | **n** |
| --- | --- |
| Systemic: Nausea | 7 |
| Headache | 7 |
| Myalgia | 7 |
| Fever | 2 |
| Local: Cough | 11 |
| Rhonchi | 2 |
| Chest pain | 2 |
| Phlegm | 10 |
| Dyspnea | none |

**Table S2.**

|  | **Baseline** | **24h** | **48h** |
| --- | --- | --- | --- |
| **n** | 16 | 10 | 6 |
| **CRP** (mg/mL) | 2.5  (2.5-6.0) | 13  (5.0-22)* | 8.0  (2.5-30) |
| **LPC** (x 10^9^/L) | 6.0  (4.0-8.2) | 7.4  (5.7-9.4)** | 5.0  (3.9-8.8) |
| **PMN** (x 10^9^/L) | 2.8  (2.0-4.6) | 4.8  (3.5-7.6)** | 2.4  (2.0-5.7) |

*Footnote.* Median values with ranges are shown. The normal range for LPC is 4.4-10.0 x 10^9^/L and the lower detection limit for CRP is 5 mg/L, respectively, in the utilized accredited laboratory of Clinical Chemistry. Asterisks indicate statistical comparisons with baseline values (*=p<0.05, **=p<0.01, paired test).

**Table S3.**

| **BAL** | **Veh** | **E** |
| --- | --- | --- |
| **Recovery** (mL) | 106  (92-120) | 103  (80-113) |
| **Viability** (%) | 82  (62-91) | 95  (74-97)** |

*Footnote.* Median values with ranges are shown. Asterisks indicate a statistically significant difference between samples harvested from vehicle- and endotoxin-exposed bronchial segments (**=p<0.01, paired test).

**Figure S1**
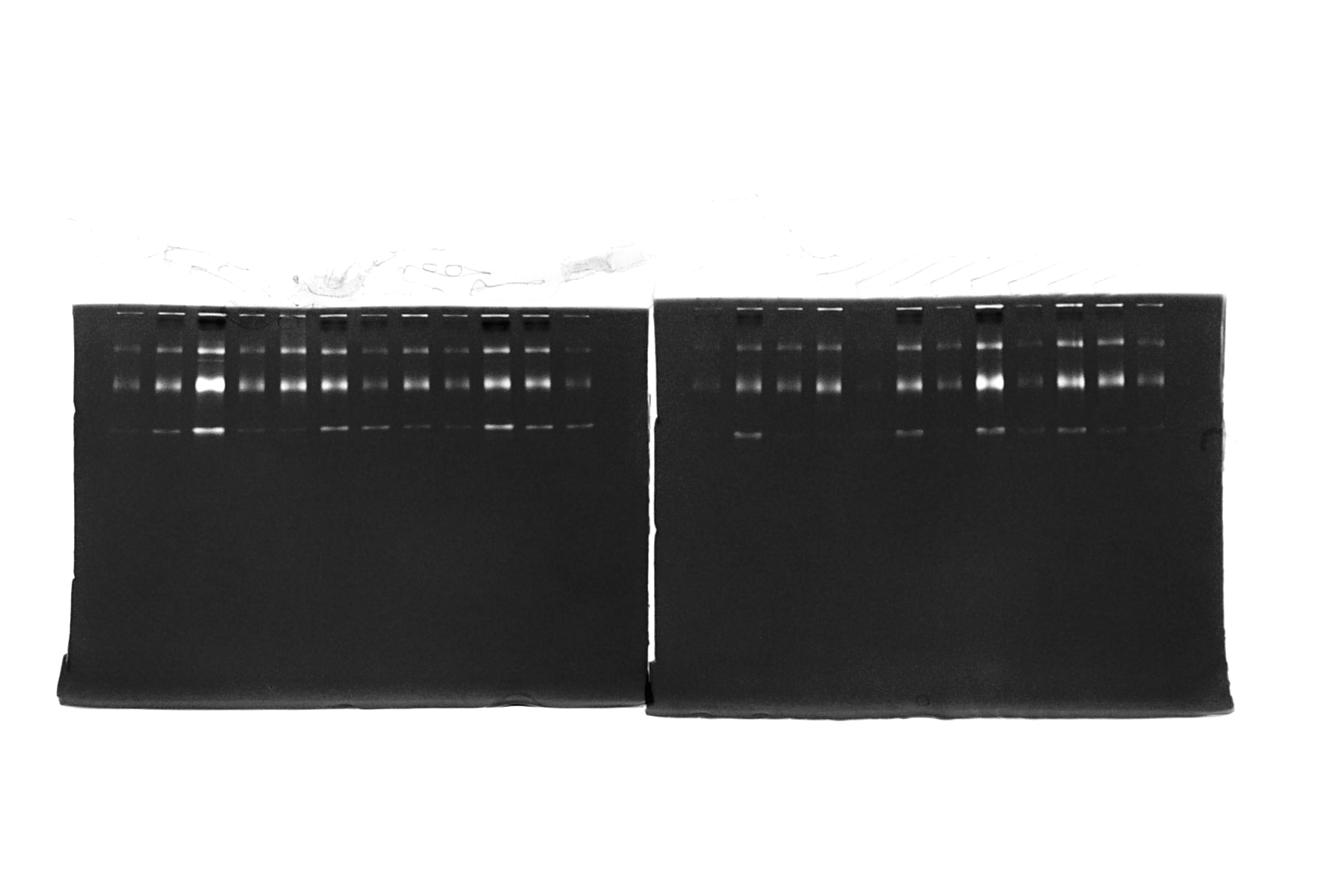


170

111

80

61

50

MMP-9 (92kDa)

MMP-2 (72kDa)

Complex bound MMP-9 (130kDa)

*

*

**Figure S2**


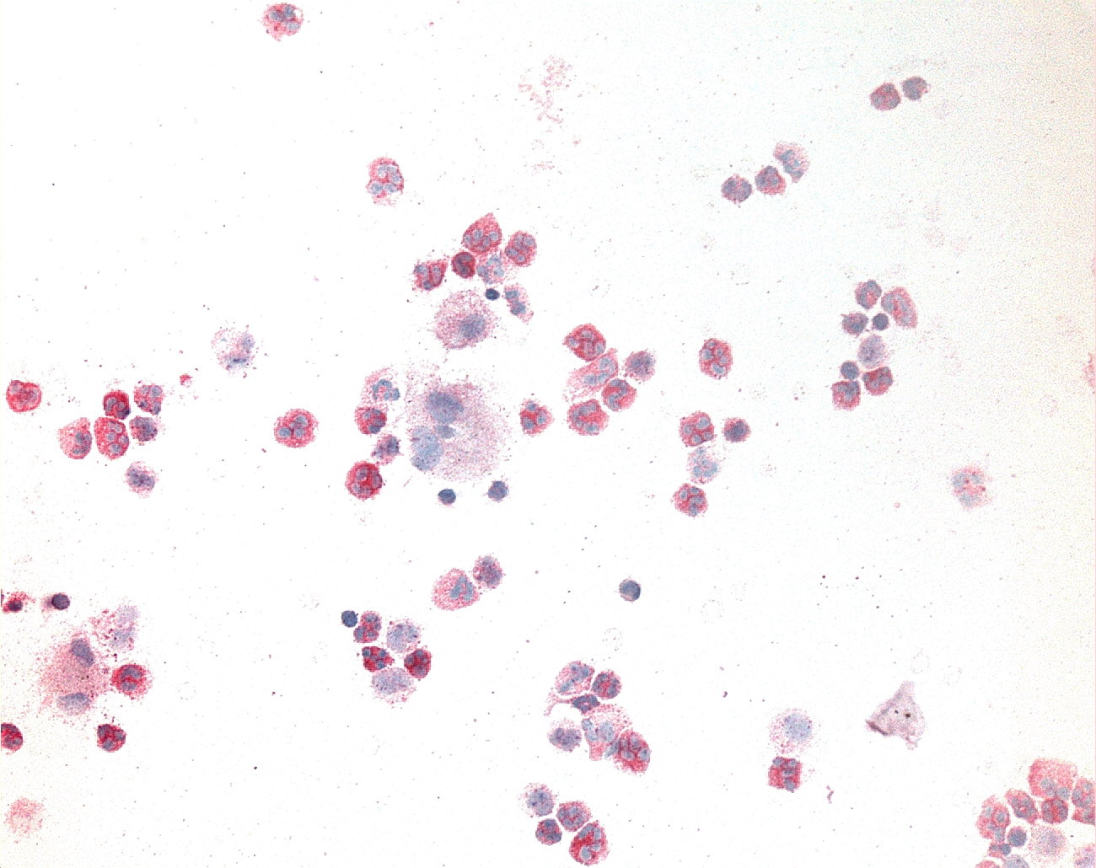
a)

b)


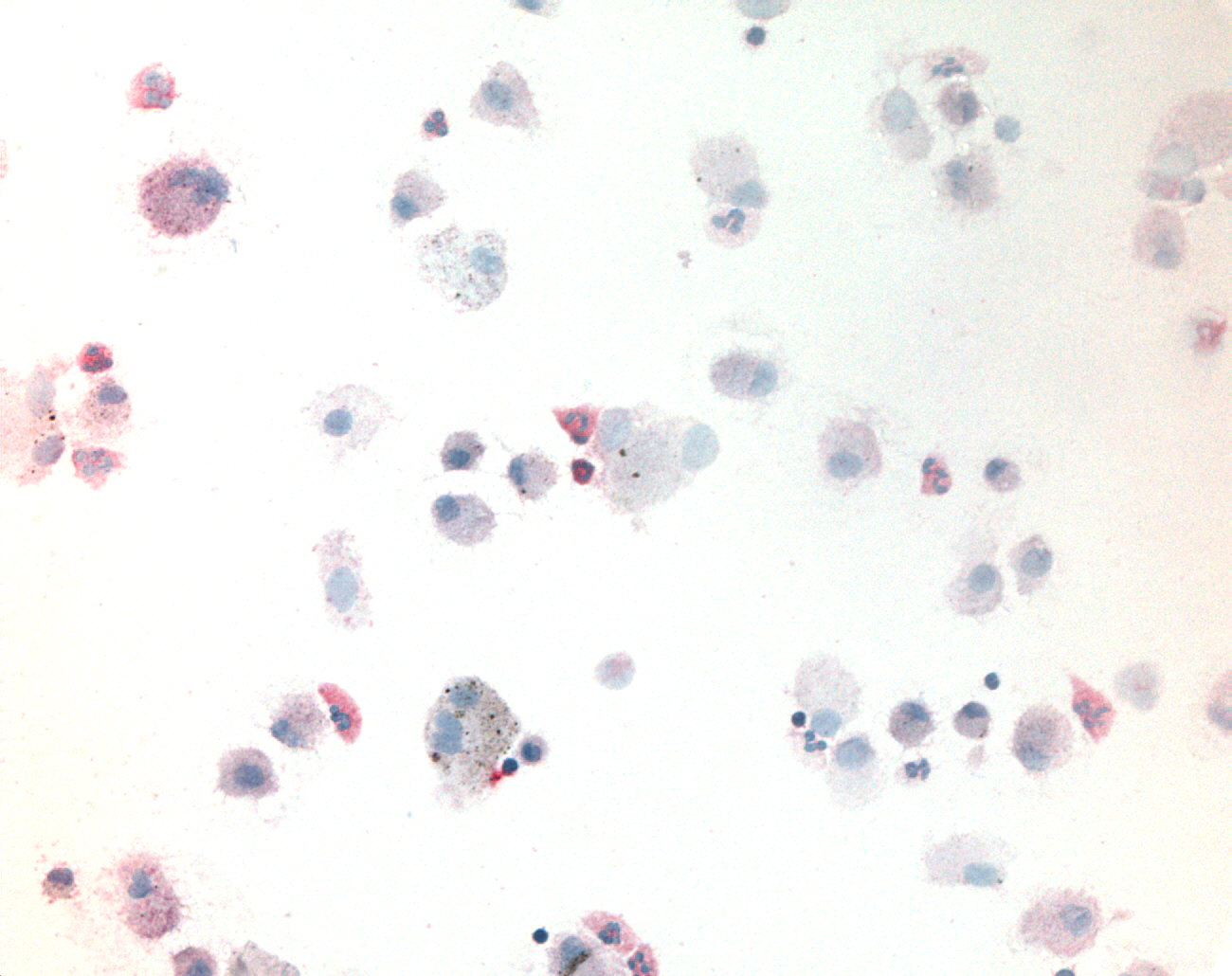


c)


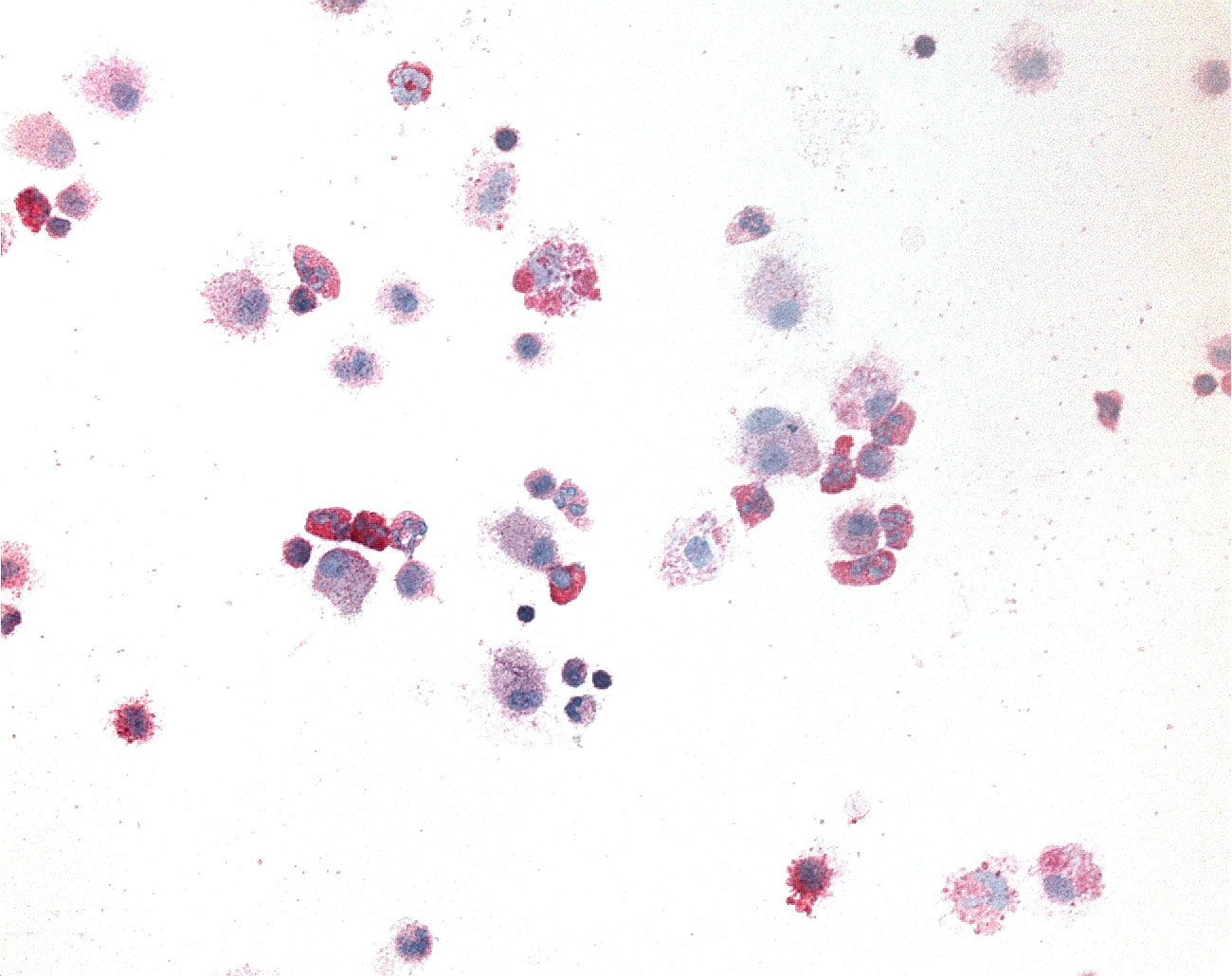

Supplement: File S1 — Table S1. Symptom assessment after the first bronchoscopy (B.I) in 18 healthy subjects. n = number of subjects reporting each symptom. Table S2. Blood concentrations of C-reactive protein (CRP), total leukocytes (LPC) and polymorphonuclear leukocytes (PMN's) at baseline prior to bronchoscopy I (B.I), and 24 or 48 hours (h) after intra-bronchial endotoxin exposure in healthy subjects. Table S3. Recovery of and cell viability in bronchoalveolar lavage (BAL) fluid samples harvested after intra-bronchial exposure to vehicle (Veh) and endotoxin (E), respectively, in 18 healthy subjects. Samples were harvested either 24 (n = 11) or 48 (n = 7) hours after the exposure. Figure S1. Photo of representative zymography gel, based upon analysis of cell-free bronchoalveolar lavage (BAL) fluid samples harvested after intra-bronchial exposure to vehicle (no arrow) and endotoxin (arrow), respectively, at 24 hours (h, black arrow) or 48 h (open arrow) in healthy subjects. The samples display three bands representing different molecular weights, corresponding to matrix metalloproteinase (MMP)-2 (72 kD), MMP-9 (92 kD) and a complex bound form of MMP-9 (130 kD). *Two lanes from the same subject. Figure S2. Photomicrograph of representative cytospin slides showing bronchoalveolar lavage (BAL) cells containing intracellular matrix metalloproteinase (MMP)-9 harvested after intra-bronchial exposure to endotoxin and vehicle, respectively, in healthy subjects. Immunoreactivity to MMP-9 is red and polymorphonuclear (PMN) cells with positive staining are indicated with arrows. Panels show samples from segments exposed to a) endotoxin and b) vehicle from the same subject at 24 hours (h) and c) endotoxin at 48 h after exposure from another subject. In panel c, the PMN indicated by an arrow is engulphed by a macrophage. (DOCX) [file pone.0075032.s001.docx]
